# *Kodamaea ohmeri* fungemia in severe burn: Case study and literature review

**DOI:** 10.1016/j.mmcr.2018.07.005

**Published:** 2018-07-20

**Authors:** Ayaka Tashiro, Takahito Nei, Ryoji Sugimoto, Akiko Watanabe, Jun Hagiwara, Toru Takiguchi, Hiroyuki Yokota, Katsuhiko Kamei

**Affiliations:** a Department of Clinical Laboratory, Nippon Medical School Hospital, Tokyo 113-8603, Japan; b Department of Infection Control and Prevention, Nippon Medical School Hospital, Tokyo 113-8603, Japan; c Department of Emergency and Critical Care Medicine, Nippon Medical School Hospital, Tokyo 113-8603, Japan; d Medical Mycology Research Center, Chiba University, Chiba 260-8673, Japan

**Keywords:** *Kodamaea ohmeri*, Echinocandin, Fungemia, Microbial substitution

## Abstract

*Kodamaea ohmeri* is a relatively rare yeast isolated form clinical specimens, and it is known to be a causative fungus of severe invasive infectious diseases in immunocompromised hosts. Herein, we describe fungemia due to *K. ohmeri* in a patient with a severe extended burn. The isolate was obtained from not only blood specimens but also skin lesions. We should be aware of risk for fungemia including *K. ohmeri* in case of severe burn.

## 1 Introduction

*Kodamaea ohmeri* is relatively rare yeast belonging to be ascomycete group, previously known as *Pichia ohmeri* or *Yamadazyma ohmeri* [1]. Now, genus *Kodamaea* is divided into 5 species and only *K. ohmeri* shows pathogenicity in humans [1]. However, systemic infection due to *K. ohmeri* is generally considered to be rare. Recently, *K. ohmeri* was recognized as an important pathogenic fungus in immunocompromised hosts. Herein, we describe a patient with a severe extended burn and *K. ohmeri* fungemia, suspected to be associated with skin colonization.

## 2 Case

### 2.1 Case description

A female in her 60 s with a severe extended burn was referred to our emergency department　from a suburban hospital (day 0). The burn extent was evaluated as follows; 12% area of 2nd degree (deep dermal) burns to the four extremities and 45% area of 3rd degree burns to the lower jaw, thorax and abdomen, as well as both lower extremities from the pubic region. Cefazolin sodium was administered (2 g every 8 h) as prophylaxis against skin soft tissue infection (day 0). In addition, she had undergone debridement of the precordium, abdomen and both lower extremities, and the 1st autograft was performed on the day 2.

We continued treating her in our intensive care unit. However, *Pseudomonas aeruginosa* and *Enterobacter cloacae* were isolated from a wound lesion (day 6). We thus switched the antimicrobial agent to tazobactam piperacillin (2.25 g every 6 h, from day 8) after performing another autograft (day 6). Despite ongoing intensive therapy, the skin soft tissue infection did not resolve. Subsequently, we isolated *E. cloacae* by blood culture (blood sample obtained at day 7), and her condition gradually deteriorated. The wound lesion showed no resolution, and not only *E. cloacae* but also *K. ohmeri* and *Candida krusei* were isolated on the day 14 from the wound lesion and blood. We continued administration of anti-pseudomonas antimicrobial agents and added an antifungal agent (day 15), but she progressed to sepsis.

Echinocandin (micafungin) was selected as the primary antifungal therapy (150 mg every 24 h, from day 15). However, susceptibility test using a commercial susceptibility detection kit performed in our hospital laboratory suggested a low susceptibility of *K. ohmeri* isolate to micafungin. Moreover, the susceptibility results were obtained on the day 19, five days after the recognition of fungemia. Though we switched treatment to liposomal amphotericin-B (3 mg/kg every 24 h, from day 25), her condition removed grave. Despite administering antimicrobials and antifungal agent, we isolated *Enterococcus faecium*, *Candida krusei*, and *Kodamaea ohmeri* from blood obtained on the day 25. On the day 29, metabolic acidosis became uncontrollable, and she died on the day 31.

### 2.2 Microbiological study

**Fig. 1:**
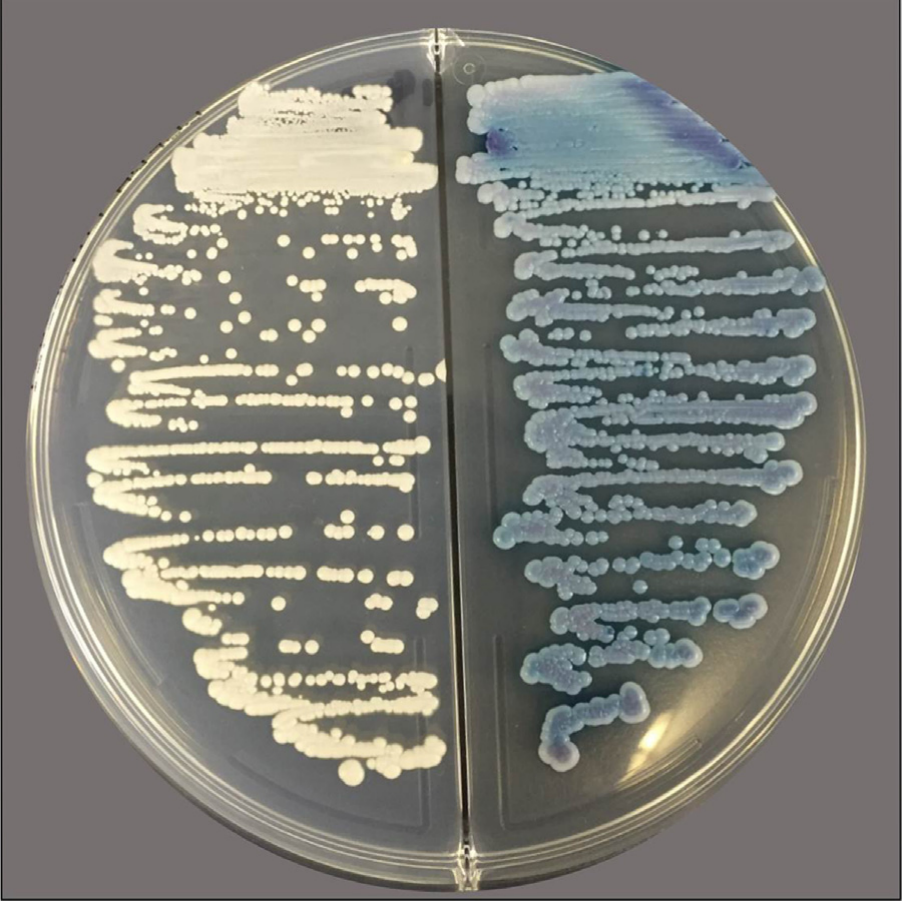
Isolation of colonies from blood culture with potato dextrose agar (left) and CHROMagr Candida® media. White and pale-peach colored, respectively, colonies were observable at 24 h. However, these later became blue at 72 h of culture.

We used BACTEC^TM^ Plus Aerobic/F Culture Vials and anaerobic/F Culture Vials (Bekton Dickinson and Company, Tokyo, Japan) for blood culture sampling and incubation (BACTEC^TM^ FX, Bekton Dickinson) at 35 °C. Both aerobic/anaerobic media yielded positive results within 48 h of starting the incubation and yeast-like fungi were detected by Gram staining. For identification, we used 5% sheep serum Agar (Eiken, Tokyo, Japan), McConky Agar (Oriental Yeast Co. Ltd, Tokyo, Japan), and CHROMager® Candida/potato-dextrose Agar (Bekton Dickinson) at 35 °C in an aerobic incubation. We isolated approximately 1 mm in diameter white and pale peach colored colonies in 5% sheep serum agar and CHROMager® Candida/potato-dextrose Agar within 48 h of culture, respectively. To obtain a further evidence for identification of the organism, we used commercial identification kits, API20C^TM^ AUX and API32C^TM^ ID (Sysmex biomerieux, Tokyo, Japan), which indicated *K. ohmeri*. Moreover, it was observed that the color of the colony in CHROMager® Candida media turned blue with 72 h of culture (Fig. 1). We highly suspected the isolates to be *K. ohmeri* based on results obtained with the commercial identification kits and the color change of the colonies. We ultimately identified the strain as *Kodamaea ohmeri* using genomic molecular identification based on sequence analysis of the internal transcribed spacer (ITS) 1 + 2 regions of the ribosomal DNA based on standard protocol [2]. Basic Local Alignment Search Tool (BLAST) analysis of the ITS 1 + 2 (76 bp + 86 bp) and 5.8S ribosomal DNA (rDNA) (156 bp) sequence of the isolates exhibited 100% identity with reference strain DQ681362, EF190226, EF190229, EF192218, EF192219, EF192221, EF196810, EF196811, EF197806, EF198002, EF198005, EF198010, EF198012, EF199745, EU569326, JQ806377, KY178312 and LC317627 (accession number). On the other hand, the homology with type strain (CBS 5367, ATCC 46053, CCRC 22178, IFO 1271, NRRL Y-1932, UCD 75–73, and WM 807) was 99.7% (317 bp/318 bp, including 5.8 S rDNA).

We determined susceptibilities to antifungal agents by broth microdilution based on Clinical and Laboratory Standards Institute (CLSI) M27-A3 methods [3]. The results are shown in Table 1.

**Table 1:** Antifungal agents: Isolate susceptibilities.

Antifungal agents	Present strain^a^	Ref 4.	Ref 18.	Ref 19.	Ref 20.
Strain number		13	2	38	2
Amphotericin-B	**1**	0.25–0.5	0.125–0.5	0.25–1	0.5
Fluconazole	**8**	2–32	4-> 128	0.5–64	16–32
itraconazole	**0.25**	0.125–0.5	0.25–0.5	0.06–4	0.25–1
Voriconazole	**0.06**	0.03–0.5	0.015–0.5	0.03–8	0.06–0.25
Caspofungin	**0.12**	0.125–0.25	ND	0.12–1	> 16
micafungin	**0.03**	0.03–0.06	ND	ND	ND
5-flucytosine	**0.5**	ND	ND	ND	0.06–0.12

a Present strain was measured by broth microdilution method based on CLSI M27-A3 using IC (inhibitory concentration) 50 as an endpoint, except for amphotericin-B (IC100). The unit of all numerical data is μg / ml.

## 3 Discussion

We can identify most yeast-like fungi based on their fermentations and hydrocarbon assimilation as well as characteristic features on morphology including colony appearance. Like other fungi, *K. ohmeri* is also identifiable by these methods. Thus, commercial based identification is widely available to identify *K. ohmeri*. Among notable characteristics, colony color change on chromogenic media is useful for identification. This method employs a commercially available isolation medium, CHROMagr® Candida, and the colony changes color from pale-pink to blue with 72 h of incubation [4], [5]. We recognized this color change when using the identification kit for isolation. On the other hand, commercial isolation kits do not have sufficient sensitivity and specificity for detection. Thus, we consider confirmation of colonies with the naked eye to be important, along with biochemical identification. Among the yeast-like fungi identified, *Candida parapsilosis* and *C. guillermondii* can easily be mistaken from each other in the early stage of culture, and *C. tropicalis* colonies show a similar color change after 72 h. *K. ohmeri* should be distinguished from similar organism using every available method. At present, *Candida auris* is attracting attention globally as a multi-drug resistant strain [6], [7]. Previous reports have shown that commercial identification kits can misread *C. haemulonii* as *K. ohmeri* [4], and *C. auris* cannot be distinguished from *C. haemulonii* with conventional identification methods. To our knowledge, there are no previous reports describing misidentification *C. auris* with *K. ohmeri*.

*K. ohmeri* had been recognized as a fungal contaminant but not as being pathogenic. However, since the first report of a case with sepsis due to *K. ohmeri*, case reports of invasive infectious diseases due to *K. ohmeri* have gradually accumulated. Recently, *K. ohmeri* was established as one of the important fungi causing invasive infections in humans [8]. Sepsis or fungemia [4], [5], [8], [9], [10], [11], [12], [13], [14], [15], [16], [17], [18], catheter-related blood stream infection [10], [17], phlebitis [9], peritonitis [19], and endocarditis [14], [16] have been reported. Diabetes mellitus, malignant tumor, prosthetic heart valves, and chronic kidney diseases are considered to be risk factors for invasive infections due to *K. ohmeri*. Moreover, premature infants are also considered to be at risk for invasive infections [5]. An outbreak was also reported previously [20].

Our patient was at high risk for invasive infections including those due to fungi, because she had undergone several autografts and received intravenous hyperalimentation through a central venous catheter. As noted above, *Kodamaea* fungemia usually occurs in patients with not only diabetes mellitus or an immunocompromised status but also as catheter-related bloodstream or prosthetic-related infection. On the other hand, the skin barrier completely fails in patients with severe burns, allowing bloodstream or deep organ tissue invasion by resident flora of the skin. Thus, we suspected that there might be a relationship between pathogenic microbes or fungi and resident flora of the skin, though definitive proof is lacking. Furthermore, *K. ohmeri* was not isolated from the skin prior to autografting. Therefore, we cannot confirm *K. ohmeri* to be among the resident skin flora. Indeed, *K. ohmeri* was not isolated from none of other patients at that time in our intensive care unit. Evaluating the sporadic onset of fungemia in our case would be of interest, though she died of sepsis due to several pathogenic microbes.

We also measured the susceptibility of one isolate to antifungal agents using the commercial kit based on the colorimetric method as indicated by the manufacture's manual, and found the minimum inhibitory concentration (MIC) of micafungin (above 16 μg/ml) to be relatively high. However, we confirmed the MIC for micafungin to be 0.03 μg/ml by the broth microdilution method which was recommended by CLSI (M27-A3). Though CLSI did not establish the breakpoint to judge susceptibilities for *Kodamaea* spp., most strains appear to have susceptibility with low MIC levels based on previously reported results. In fact, this initial susceptibility reports strongly influenced our choice of antifungal agent to administer. Thus, we decided that validation of the antifungal susceptibility test results was required.

In conclusion, while infection by *K. ohmeri* is rare, we should be aware that invasive infections can develop in immunocompromised hosts and other patients with risk factors. We advocate awareness of this rare fungus, as a potential cause of severe sepsis, such as in patients with severe burns, and that antifungal susceptibility test results validated for rare ascomycetous yeasts.
